# Clinical Instruction

**Published:** 1854-09

**Authors:** 


### EDITORIAL.

### *Clinical Instruction.*

"But in the United States, *hospitals are so few*, comparatively, and conducted in such a manner, that only a *small portion* of medical students can expect to have access to them. Hence, the deficiency must be made up some other way, and the only means for supplying this deficiency must be found in the office of the private preceptor. For the amount of real and useful clinic instruction, derived from clinical lectures and public surgical operations performed in the amphitheatre of our medical colleges, is very small indeed; and also a great portion of that derived from the amphitheatre of the American hospitals is not much beter; and what is obtained, is mostly on *chronic* diseases, leaving those of an acute character, with which the country practitioner has most to do, almost wholly unnoticed."

The above paragraph is from an essay on Medical Education, presented to the Medical Association of Southern Central New York. The same ideas were advanced by Dr. Z. Pitcher, of Detroit, in his Report on Medical Education, read to the American Medical Association in May, 1853; and similar representations in reference to *American* clinical instruction, have been so frequently advanced in essays and public addresses, that it may not be amiss to examine the subject briefly. That medical students and teachers in this country still pay too little attention to clinical instruction, we will freely admit. This, however, is not owing so much to the *small number* of hospitals, or the manner in which they are managed, as it is to the want of any adequate appreciation of the importance of this department of medical instruction, on the part of students and the profession generally. For instance, there are permanent and well-filled hospitals in Boston, New York, Pniladelphia, Baltimore, Charleston, New Orleans, Cincinnati, Chicago, Buffalo. and several other cities; making a *number* amply sufficient to accommodate every student in the United States who is prepared by previous study to profit by attending them. Neither is it true that the clinical instruction capable of being given in the hospitals of this country "is mostly on *chronic diseases*, leaving those of an acute character, with which the country practitioners have most to do, almost wholly unnoticed."

On the contrary, by far the larger part of those received into our hospitals are laboring under the most severe forms of fever, inflammation, &c., together with serious surgical injuries and diseases. So far as we have been able to observe, the proportion of *acute* febrile and inflammatory affections in our hospitals is greater than is usually met with in private practice. Hence, if a large proportion of those who annually enter the ranks of our profession, do so without adequate clinical instruction it is not from a deficiency in the *number* of hospitals, or in the kind of disease admitted into them. The true causes must be looked for elsewhere. In the first place, a large proportion, both of students and preceptors, have no adequate idea of the importance of direct and systematic clinical instruction. The preceptors, many of them, having never received such instruction themselves, are not likely to impress its importance very strongly on the minds of their pupils.

Hence, the selection of a medical college by the student or his preceptor is made to depend almost wholly on other considerations. And among these, *pecuniary* considerations are certainly not the least influential. The great mass of medical students in this country possess limited pecuniary resources, and are compelled thereby to limit in a corresponding degree their privileges. But, by some strange want of adaptation of means to ends, or some false idea of professional dignity, the pecuniary expense required for attendance on most of those schools where good hospitals are accessible is so great, especially when compared with the expenses required at a considerable number of other schools where *no hospitals exist*, that it presents a strong barrier in the way of the student, and directly prevents that full and universal recognition of the importance of hospital or bed-side instruction, which is necessary for the welfare of the profession and the community. But, aside from all these considerations, there is, undoubtedly, throughout the hospitals of our country great defects in the arrangements for clinical teaching. In many the time devoted to the subject is not at all proportionate to its importance. For instance, in Cincinnati, Philadelphia, and other Atlantic cities, where large hospitals exist, special clinical instruction is given only twice per week, when the whole hospital class are expected to attend at the same hour. If they are admitted into the wards directly to the bed-side of the sick, their number is such, that very few of them can personally examine the patients. Consequently, they resort chiefly to the amphitheatre, where they can be seated as in a lecture-room, and view at a distance surgical operations, and *listen* to clinical instruction over such medical cases as can be conveniently moved from the wards into the room. Such clinical instruction, we admit, gives the student more shadow than substance. It is much better calculated to dazzle the eye and satisfy the mind of the inexperienced by its *show*, than to impart true skill in diagnosticating and treating diseases. And it is, doubtless, to this kind of clinical instruction that allusion is made by such writers as the one quoted at the beginning of this article.

They do injustice, however, when they represent this as the only kind of clinical institution given in American hospitals, for we believe th re are several well organized institutions, in which the time given to this branch is much more in accordance with its importance. The true object of clinical instruction should be to bring the student directly in contact with the sick so frequently that he may observe all the changes of morbid action and the effects of remedies as exhibited during the whole progress of each individual case; and it should also furnish such opportunities for manual examinations as will thoroughly train the eye, the ear, and the hand in the delicate work of physical diagnosis. We know that the practical accomplishment of these objects, requires much labor on the part of the teacher, and patience on the part of the learner.

It cannot be made a work of *show* and *eloquence*, but must be confined to patient, familiar personal instruction. Of course only a *limited* number can receive such instruction at the same hour, and around the same sick bed. And yet, by proper system and arrangement, all the students who are sufficiently advanced in medical studies could be amply accommodated in the hospitals of our country. For the last four years the students of Rush Medical College have had access to the wards of the Mercy Hospital, (formerly called the Illinois General Hospital).

The institution contains an ample supply of patients, four-fifths of whom are laboring under acute diseases, embracing all the forms of fever and local inflammations. During the College term the wards are under the charge of the Professors of Surgery and Practical Medicine, each of whom devotes an hour every morning (except Sundays) to clinical instruction in their respective wards. The students who take the hospital ticket are divided into two general classes, one of which visits the hospital each morning. When there, they sub-divide, one half entering the surgical, and the other the medical wards. When the same general class visit the hospital again, the sub-divisions interchange: those who went with the surgical profession before now go with the medical. In this way, each student taking the hospital ticket visits the hospital, and receives clinical instruction every other day, or three times a week.

And yet the number present at one time in the same ward is always so limited that all can come to the bed-side, and *are* permitted to examine the pulse, the tongue, the countenance, and to individually percuss and auscultate. Not only are they *permitted*, but *required* to do this, and also to practice on the healthy chest directly under the superintendence of the Professor of Practical Medicine, until both the ear and hand of the learner are trained to their appropriate work. By the foregoing systematic arrangements, ninety students received regular clinical instruction in the Mercy Hospital during the last College term. And we must be pardoned if we claim for it the title of "*real and useful clinical instruction*;" something more than a *mere show* over "dazzling surgical operations," and "chronic diseases." D.

